# Gabrb3 endothelial cell-specific knockout mice display abnormal blood flow, hypertension, and behavioral dysfunction

**DOI:** 10.1038/s41598-022-08806-9

**Published:** 2022-03-22

**Authors:** Anass Agrud, Sivan Subburaju, Pranay Goel, Jun Ren, Ashwin Srinivasan Kumar, Barbara J. Caldarone, Wangde Dai, Jesus Chavez, Dai Fukumura, Rakesh K. Jain, Robert A. Kloner, Anju Vasudevan

**Affiliations:** 1 Angiogenesis and Brain Development Laboratory, Huntington Medical Research Institutes (HMRI), 686 S Fair Oaks Avenue, Pasadena, CA 91105 USA; 2 Department of Psychiatry, Harvard Medical School, Boston, MA 02215 USA; 3 Division of Basic Neuroscience, McLean Hospital, 115 Mill Street, Belmont, MA 02478 USA; 4 Edwin L. Steele Laboratories, Department of Radiation Oncology, Massachusetts General Hospital and Harvard Medical School, Boston, MA 02114 USA; 5 Harvard-MIT Division of Health Sciences and Technology, Massachusetts Institute of Technology, Cambridge, MA 02139 USA; 6 Mouse Behavior Core, Department of Genetics, Harvard Medical School, Boston, MA USA; 7 Huntington Medical Research Institutes, Pasadena, CA USA; 8 Division of Cardiovascular Medicine, Department of Medicine, Keck School of Medicine at University of Southern California, Los Angeles, CA USA

**Keywords:** Cell biology, Developmental biology, Neuroscience, Diseases, Medical research

## Abstract

Our recent studies uncovered a novel GABA signaling pathway in embryonic forebrain endothelial cells that works independently from neuronal GABA signaling and revealed that disruptions in endothelial GABA_A_ receptor-GABA signaling from early embryonic stages can directly contribute to the origin of psychiatric disorders. In the GABA_A_ receptor β3 subunit endothelial cell conditional knockout (*Gabrb3*^*ECKO*^) mice, the β3 subunit is deleted selectively from endothelial cells, therefore endothelial GABA_A_ receptors become inactivated and dysfunctional. There is a reduction in vessel densities and increased vessel morphology in the *Gabrb3*^*ECKO*^ telencephalon that persists in the adult neocortex. *Gabrb3*^*ECKO*^ mice show behavioral deficits such as impaired reciprocal social interactions, communication deficits, heightened anxiety, and depression. Here, we characterize the functional changes in *Gabrb3*^*ECKO*^ mice by evaluating cortical blood flow, examine the consequences of loss of endothelial *Gabrb3* on cardiac tissue, and define more in-depth altered behaviors. Red blood cell velocity and blood flow were increased in the cortical microcirculation of the *Gabrb3*^*ECKO*^ mice. The *Gabrb3*^*ECKO*^ mice had a reduction in vessel densities in the heart, similar to the brain; exhibited wavy, myocardial fibers, with elongated ‘worm-like’ nuclei in their cardiac histology, and developed hypertension. Additional alterations in behavioral function were observed in the *Gabrb3*^*ECKO*^ mice such as increased spontaneous exploratory activity and rearing in an open field, reduced short term memory, decreased ambulatory activity in CLAMS testing, and altered prepulse inhibition to startle, an important biomarker of psychiatric diseases such as schizophrenia. Our results imply that vascular *Gabrb3* is a key player in the brain as well as the heart, and its loss in both organs can lead to concurrent development of psychiatric and cardiac dysfunction.

## Introduction

The global burden of mental health disorders reveals staggering numbers with predictions for a rapid increase in the number of individuals with a mental health problem over the next decade. From common disorders such as anxiety and depression, to complex disorders such as schizophrenia, almost one billion people worldwide suffer from a psychiatric disorder. Schizophrenia, a debilitating mental disease that affects twenty million people worldwide, is still an enigma with regard to its exact pathophysiology and potential underlying causes on a cellular and molecular level. There is a multitude of evidence that the pathogenesis of anxiety, depression, and schizophrenia are neurodevelopmental in nature^1–4^, and impairment of cortical and hippocampal GABAergic interneurons are important contributors^5–11^. Schizophrenia is characterized by a number of positive and negative symptoms which include behavioral alterations and cognitive deficits that appear during childhood and adolescence.

Several studies have reported the reduction or loss of gamma-aminobutyric acid (GABA) signaling in frontotemporal brain regions as contributing to the pathophysiology of schizophrenia^9,11–14^. The GABA_A_ receptor is responsible for the majority of the physiological actions of GABA, and lower GABA_A_ receptor availability has been associated with symptom severity^15^. GABA_A_ receptor subunits such as α5 and β2 have been reported as robust schizophrenia candidate genes^16,17^. GABA_A_ receptor β3 (*Gabrb3*) polymorphism has been reported in schizophrenic patients^18^. Additionally, lower expression of GABRB3 protein has been reported in the superior frontal cortex [Brodmann Area 9 (BA9)] of patients with schizophrenia versus controls^19^. A systematic gene expression analysis of 22 GABA related genes in postmortem superior temporal gyrus (STG) samples of both elderly and young patients with schizophrenia showed a downregulation of *Gabrb3*^20^.

Traditional views of neocortical development have depicted the source of GABA-GABA_A_ receptor signaling to be exclusively neuronal^21–24^. Recently, we uncovered a novel signaling pathway for GABA and its receptors within forebrain endothelial cells that work independently of the traditional GABA neuronal pathway^25^. This study introduced conceptual changes and produced a new way to think about the origin and mechanisms of psychiatric illnesses^26^. Several GABA_A_ receptor subunits are expressed in periventricular endothelial cells of the embryonic forebrain and include the α1, α2, α4, α5, β1, β2, β3 and γ subunits, with the β3 subunit being highly enriched during the developmental period^25,27^. Our previous work in which *Gabrb3* was deleted selectively from endothelial cells to generate the *Gabrb3* endothelial cell conditional knockout (*Gabrb3*^*ECKO*^) mouse model highlighted several interesting findings^25^. First, loss of endothelial *Gabrb3* resulted in a loss of function of endothelial GABA_A_ receptors that are activated by GABA and affected calcium transients that determine cell proliferation events (Supplementary Fig. 1a). Second, endothelial *Gabrb3* was not only essential for GABA_A_ receptor functions, but it also modulated GABA expression, resulting in lowered GABA levels during embryonic development (Supplementary Fig. 1a). Third, endothelial cell-derived GABA failed to activate a positive feedback cycle in *Gabrb3*^*ECKO*^ endothelial cells, essential for its autocrine role in angiogenesis, and paracrine role as a chemoattractant and guidance cue for long-distance GABAergic interneuron migration (Supplementary Fig. 1a). Fourth, the loss of function of the GABA_A_ receptor in the *Gabrb3*^*ECKO*^ model was specific to endothelial cells, while there was no loss of function in neuronal cells (Supplementary Fig. 1b). Fifth, the loss of functional endothelial GABA_A_ receptors, and partial loss of endothelial GABA, impaired telencephalic angiogenesis and angiogenesis guided GABAergic neuronal migration in vivo as observed in controls (Supplementary Fig. 1c, d). While possible paracrine roles of neuronal GABA on angiogenesis cannot be overlooked, it was clearly unable to rescue the vascular defects in the absence of endothelial GABA. Therefore, the consequences persisted in the adult brain reflecting as reduced vascular densities in the cingulate, motor, somatosensory, and parietal cortex, as well as a reduction of cortical interneurons (Supplementary Fig. 1e). And finally, multifaceted behavioral deficits such as impaired home cage social behaviors, grooming, increased anxiety, depression, impaired sociability, and decreased social novelty were observed that are common to many overlapping psychiatric disease categories (Supplementary Fig. 1f.)^25^. That endothelial-specific loss of *Gabrb3* from early embryonic stages can modulate key aspects of brain development and lead to behavioral dysfunction, highlighted a direct contribution of brain vasculature to the origin of psychiatric disorders^25^.

Blood flow changes have been repeatedly observed in patients with anxiety, depression, and schizophrenia^28–31^; however, this is usually associated with inflammation or changes in neural plasticity. Here we characterized our *Gabrb3*^*ECKO*^ mice further with regard to blood flow and performed an in-depth behavioral analysis. We show how prenatal loss of endothelial *Gabrb3* can directly lead to abnormal increases in neocortical blood flow and contribute to dysfunction in several behavioral domains that have been linked with anxiety, depression, and schizophrenia. Additionally, the *Gabrb3*^*ECKO*^ mice developed hypertension, suggesting that vascular *Gabrb3* plays major roles in both the brain and the heart, and its loss in both organs may contribute to concurrent development of psychiatric illness and heart disease.

## Results

### Increased blood flow in capillaries in the ***Gabrb3***^***ECKO***^ neocortex

**Figure 1:**
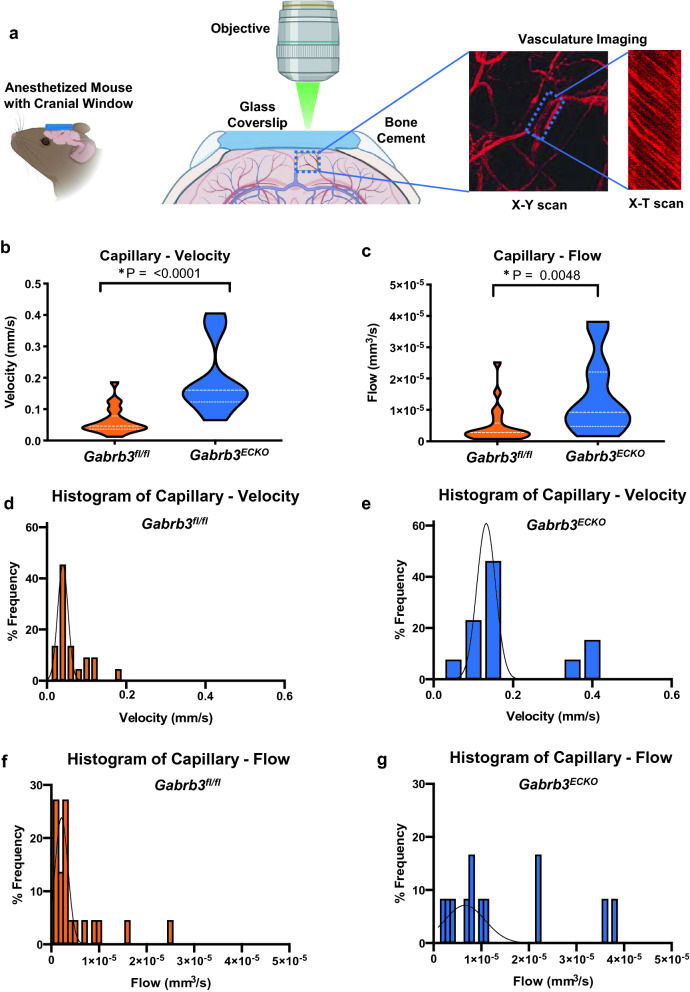
Blood flow changes in *Gabrb3*^*ECKO*^ mice. (**a**) Schematic of blood flow velocity acquisition by MPLSM. Using TAMRA–dextran contrast enhanced angiography; region of interest is first identified. Then, line scan images along the central axis of individual blood vessels (x-t) are acquired. Dark streaks (negative contrast) correspond to RBCs moving along the central axis of the blood vessel. The slopes of these streaks correspond to the RBC velocities. Data are presented in violin plots (*P < 0.05, Student's t-test). (**b**) Violin plot showing distribution of RBC velocities in capillaries (n = 22, n = 13 vessels, respectively). (**c**) Violin plot showing distribution of blood flow in capillaries of *Gabrb3*^*fl/fl*^ and *Gabrb3*^*ECKO*^ mice (n = 22, n = 12 vessels, respectively). (**e–g**) Histograms of RBC velocity and blood flow in the capillaries in the *Gabrb3*^*fl/fl*^ versus *Gabrb3*^*ECKO*^ mice.

Previously, in the *Gabrb3*^*ECKO*^ mice, we reported a reduction in vascular densities in the embryonic telencephalon that persisted in the adult neocortex, and an increase in vessel diameters indicative of functional changes^25^. Therefore, we evaluated red blood cell (RBC) velocity and blood flow in the cerebral cortex of *Gabrb3*^*fl/fl*^ and *Gabrb3*^*ECKO*^ mice using multiphoton laser-scanning microscopy (MPLSM)^32–34^ through a cranial window (Fig. 1a). RBC velocity was significantly increased in capillaries in *Gabrb3*^*ECKO*^ mice when compared to *Gabrb3*^*fl/fl*^ mice (Fig. 1b; Supplementary Fig. 2a). Consistently, blood flow in capillaries was significantly increased in *Gabrb3*^*ECKO*^ mice when compared to *Gabrb3*^*fl/fl*^ mice (Fig. 1c; Supplementary Fig. 2b). Histograms depict the changes in RBC velocity and blood flow distribution in capillaries between the two groups (Fig. 1d-g).

### Hypertension and abnormal cardiac pathology observed in *Gabrb3*^*ECKO*^ mice

**Figure 2:**
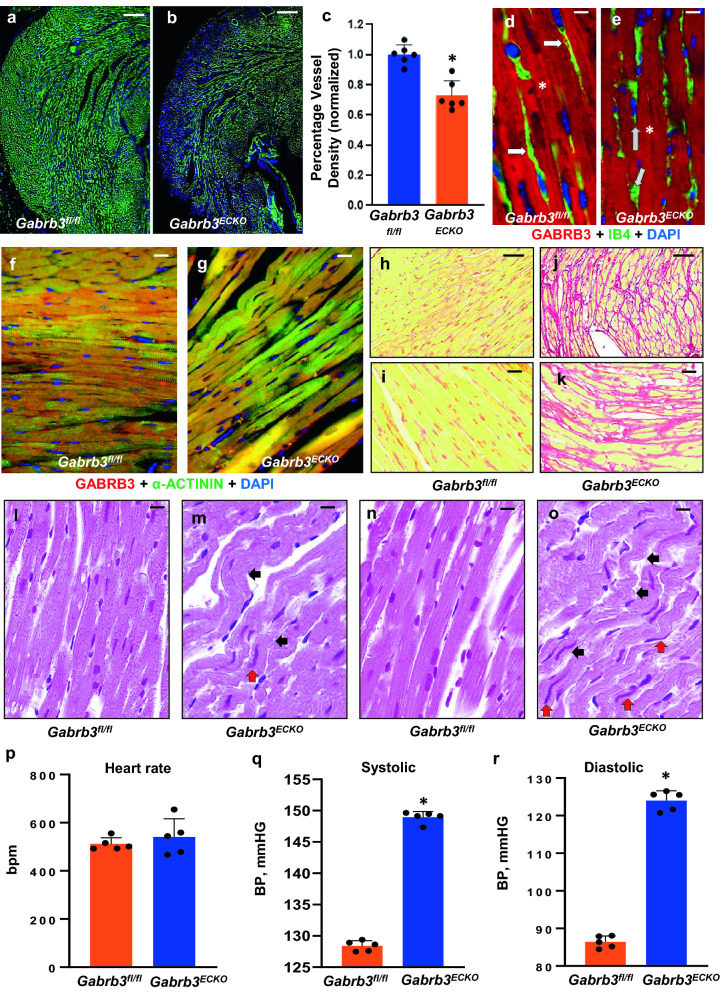
*Gabrb3*^*ECKO*^ mice have abnormal cardiac pathology and hypertension. (**a–c**) Isolectin B4-labeled vessels were significantly reduced in *Gabrb3*^*ECKO*^ hearts, when compared to floxed controls (**a**, **b**). Vessel quantification depicted in (**c**); Data represent mean ± SD (n = 6, *P < 0.05; Student's t-test). (**d**, **e**) Co-labeling with Isolectin B4 (red) and GABRB3 (green) revealed expression of GABRB3 in *Gabrb3*^*fl/fl*^ vessels only (merged in yellow, white arrows, (**d**) and its lack thereof in *Gabrb3*^*ECKO*^ vessels (gray arrows, **e**). Prominent expression of GABRB3 was observed in cardiomyocytes in both groups (asterisk). (**f**, **g**) Validation of GABRB3 expression in cardiomyocytes by co-labeling with GABRB3 and α-ACTININ in *Gabrb3*^*fl/fl*^ and *Gabrb3*^*ECKO*^ mice. (**h**–**k**) Low (**h**, **j**) and high (**i**, **k**) magnification images of normal picrosirius red staining in *Gabrb3*^*fl/fl*^ cardiac tissue (**h**, **i**), and an increase in collagen deposition, observed in *Gabrb3*^*ECKO*^ cardiac tissue (**j**, **k**). (**l-o**) H & E staining shows wavy myocardial fibers (black arrows, **m**, **o**) with long ‘worm-like’ nuclei (red arrows, **m**, **o**) in *Gabrb3*^*fl/fl*^ cardiac tissue, which is not seen in controls (**l**, **n**). (**p**) No changes in heart rate were observed in *Gabrb3*^*fl/fl*^ and *Gabrb3*^*ECKO*^ mice. (**q**, **r**) A significant increase in systolic and diastolic blood pressure was observed in *Gabrb3*^*ECKO*^ mice versus *Gabrb3*^*fl/fl*^ mice. Data represent mean ± SD (n = 5, *P < 0.05, Student's t-test). Scale bars: (**a**), 100 μm (applies to **b**, **h**, **j**); **d**, 50 μm (applies to **e**, **f**, **g**, **i**, **k**, **l-o**).

GABA and its receptors are known to be highly enriched in the CNS, both during the developmental and adult phase, with highly versatile functions, but its role in other organs such as the heart is understudied. There are recent reports of GABA_A_ receptor expression, including *Gabrb3* in adult cardiac tissue^35,36^. GABA synthesis and release by aortic endothelial cells have also been reported^37^. However, the functional significance of this GABA and its receptors expression in the adult heart remains unknown. Therefore, we evaluated whether the loss of endothelial *Gabrb3* in our model had consequences for cardiac structure and function. Labeling with isolectin B4 revealed a significant reduction in microvascular densities in adult *Gabrb3*^*ECKO*^ cardiac tissue when compared to controls (Fig. 2a-c). GABRB3 expression was observed in the microvasculature but was more prominent in cardiomyocytes in control hearts (Fig. 2d). GABRB3 expression was detected only in cardiomyocytes in *Gabrb3*^*ECKO*^ tissue and not in the vasculature as expected (Fig. 2e). GABRB3 expression in cardiomyocytes was confirmed by co-labeling with GABRB3 and α-ACTININ in control and *Gabrb3*^*ECKO*^ groups (Fig. 2f, g), and it was found to be robustly expressed in both groups.

Next, we evaluated cardiac histology by picrosirius red, and hematoxylin and eosin (H&E) staining. An increase in collagen fibers was observed by picrosirius red staining in *Gabrb3*^*ECKO*^ versus *Gabrb3*^*fl/fl*^ cardiac tissue (Fig. 2h-k), that is characteristic of cardiac fibrosis. An increase in collagen deposition can make important contributions to the deterioration of cardiac stiffness and contribute to vulnerability in the electrical and mechanical properties of the heart. H & E staining prominently revealed wavy cardiomyocytes in *Gabrb3*^*ECKO*^ mice, along with long ‘squiggly’ nuclei, which were not observed in controls (Fig. 2l-o). Wavy myocardia fibers are indicative of myocardial ischemia, and believed to be induced due to an increase in hydrostatic pressure from interstitial edema, that squeezes and stretches the neighboring fibers^38^.

To evaluate functional changes, we used the CODA tail-cuff system^39^ to measure blood pressure in *Gabrb3*^*fl/fl*^ and *Gabrb3*^*ECKO*^ mice. While no differences were observed in heart rate between the two groups (Fig. 2p), a significant increase in systolic and diastolic blood pressure was observed in *Gabrb3*^*ECKO*^ mice when compared to controls (Fig. 2q, r), indicative of hypertension. Collectively, these results highlight the far-reaching consequences of vascular *Gabrb3* elimination on the heart and suggest that loss of a common molecule *Gabrb3* from the vasculature of the brain and the heart, can contribute concurrently to brain–heart dysfunction.

### Impairment in multiple behavioral domains in ***Gabrb3***^***ECKO***^ mice

*Gabrb3*^*ECKO*^ mice showed several abnormal behaviors^25^. For instance, they showed severe grooming and poor nest building behavior in both normal and enriched environments when compared to *Gabrb3*^*fl/fl*^ mice, indicative of heightened stress/anxiety and impaired home cage social behavior^25^. In the light-dark box text, *Gabrb3*^*ECKO*^ mice showed an aversion to brightly lit open space and preferred the dark area, indicative of increased anxiety^25^. *Gabrb3*^*ECKO*^ mice showed longer periods of immobility compared to control mice in a tail suspension test, indicative of depression^25^. In a three-chamber social communication test, *Gabrb3*^*ECKO*^ mice showed no preference for a stranger mouse and spent a similar time in investigating the stranger mouse versus an inanimate object signifying impaired sociability^25^. These observations resemble negative symptoms observed in many psychiatric disorders including schizophrenia. They provided evidence that the negative valence systems and the systems for social processes were affected in the *Gabrb3*^*ECKO*^ mice. In order to further characterize the specific nature of the behavioral abnormality in the *Gabrb3*^*ECKO*^ mice, we performed additional behavioral tasks to assay the cognitive and the somatosensory systems.

### Open field locomotor activity

**Figure 3:**
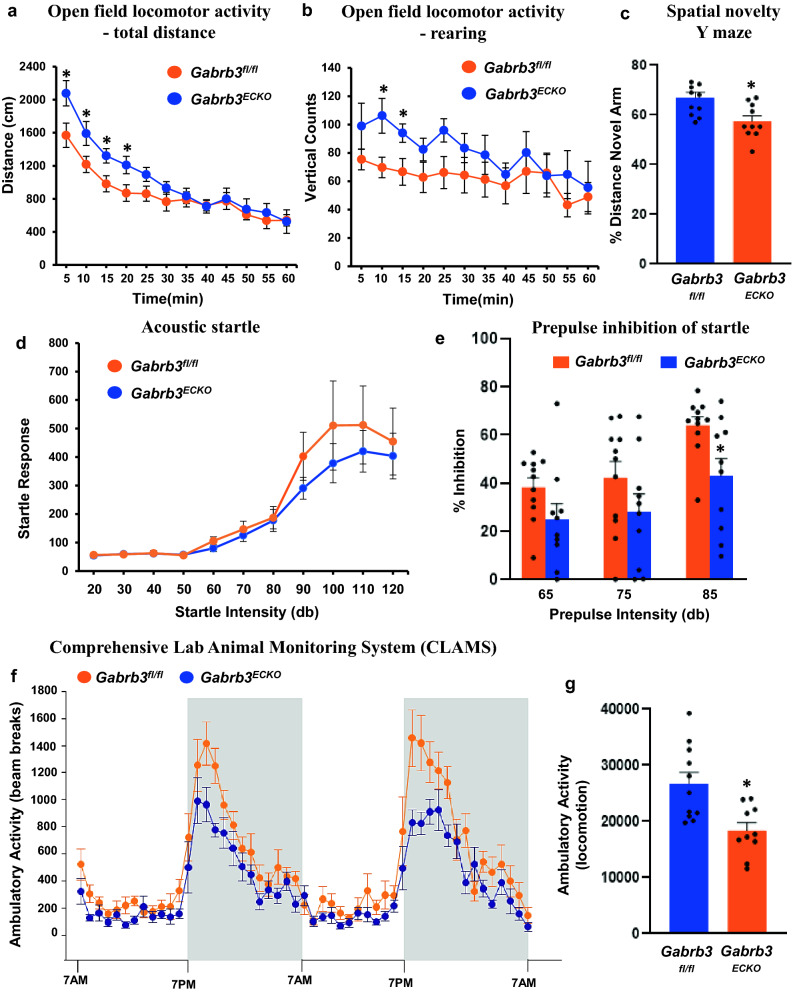
Behavioral characterization of *Gabrb3*^*ECKO*^ mice. (**a**, **b**) *Gabrb3*^*fl/*fl^ (n = 11) and *Gabrb3*^*ECKO*^ (n = 10) mice were placed into the center of a locomotor activity chamber to assess spontaneous exploratory activity. Exploration time was for a total of 60 min. The mouse’s movements were tracked and recorded automatically. Total distance covered (**a**) as well as rearing events (**b**) were analyzed separately. Data represent mean ± SEM, *P < 0.05, ANOVA. (**c**) *Gabrb3*^*fl/fl*^ (n = 11) and *Gabrb3*^*ECKO*^ (n = 10) mice were tested in a Y maze to assess short term memory for a familiar and novel place. Mice were video recorded, and the time spent in the previously accessible arm (i.e., familiar arm) and the previously blocked arm (i.e., novel arm) was measured during the free choice trial. For each subject, the % distance exploring the novel arm during the free choice trial was calculated using the formula: % novel = novel/(novel + familiar time) × 100. Data represent mean ± SEM, *P < 0.05, ANOVA. (**d**) *Gabrb3*^*fl/fl*^ (n = 11) and *Gabrb3*^*ECKO*^ (n = 10) mice were given 110 trials of startle pulses of various intensities ranging from variable 20–120 dB. Startle responses for each stimulus intensity were averaged. (**e**) Percent inhibition of acoustic startle (120 dB) following three prepulse intensities (65, 75, or 85 dB) in *Gabrb3*^*fl/fl*^ (n = 11) and *Gabrb3*^*ECKO*^ (n = 10) mice are shown. Data represent mean ± SEM, *P < 0.05. (**f**) Locomotor activity of *Gabrb3*^*fl/fl*^ (n = 11) and *Gabrb3*^*ECKO*^ (n = 10) mice was measured for 4 days using CLAMS. Data are presented for the last 48 h (7 a.m.-7 p.m.) of the 4-day period. (**g**) The sum of the counts over the last two days from ‘f’ is quantified here. Data represent mean ± SEM, *P < 0.05, ANOVA.

One of the comorbidities of psychiatric diseases is hyperactivity either at baseline or in response to the mild stress of a novel environment^40^. Some schizophrenic patients exhibit “psychomotor agitation” which includes increased stereotypic movements as well as hyperactivity. Locomotor activity or a locomotor response to a novel environment might mimic this psychomotor agitation. In an open field paradigm, analysis of the total distance travelled resulted in a significantly higher locomotor activity for *Gabrb3*^*ECKO*^ mice at the start of the session (Fig. 3a). There was a tendency of the *Gabrb3*^*ECKO*^ mice to show more significant rearing than their control counterparts (Fig. 3b), indicative of behavioral stereotypy^41^.

### Cognitive systems

Cognitive impairment in psychiatric disease manifests as deficits in information processing, abstract categorization, executive function, cognitive flexibility, attention, memory, and visual processing^42,43^. Previously we had used the tube dominance test to assess cognition in *Gabrb3*^*ECKO*^ mice, in particular social dominance through measurement of aggression, and *Gabrb3*^*ECKO*^ mice had shown fewer wins in comparison to controls^25^. Importantly, *Gabrb3*^*ECKO*^ mice have a reduction in hippocampal size^25^. A reproducible cognitive deficit in psychiatric diseases such as schizophrenia is an impairment of working memory^42,44^. Therefore, we used the spatial novelty Y maze, to measure spatial-working memory, a hippocampus-dependent trait^45^. Both *Gabrb3*^*fl/fl*^ and *Gabrb3*^*ECKO*^ mice learned the task. However, the percent distance covered in the novel arm was significantly lower for the *Gabrb3*^*ECKO*^ mice when compared to the control mice (Fig. 3c) indicative of impaired cognitive functions. No significant differences were detected between *Gabrb3*^*ECKO*^ and *Gabrb3*^*fl/fl*^ groups in the contextual and cued fear conditioning paradigm (Supplementary Fig. 3), that assesses hippocampal and amygdala-dependent learning and memory performance in rodents.

### Acoustic startle

Deficits in sensorimotor gating include altered reactions to prepulse-inhibition^46,47^. In *Gabrb3*^*ECKO*^ mice, responses to acoustic startle showed an increasing trend at higher startle intensities than in *Gabrb3*^*fl/fl*^ mice, but the response did not significantly differ between the two groups at startle intensities from 20 to 120 dB (Fig. 3d).

### Prepulse inhibition (PPI)

An altered prepulse inhibition to startle is considered an important biomarker for psychiatric diseases such as schizophrenia^48^. Pulses of sound, presented just prior to a startle stimulus, resulted in the dampening of the subsequent startle response in both control and knockout mice. The percentage of inhibition showed a decreasing trend in *Gabrb3*^*ECKO*^ mice when compared to controls and was significantly decreased at 85 dB intensity (Fig. 3e). These findings indicate sensorimotor gating deficits, and an impairment of the somatosensory system in the *Gabrb3*^*ECKO*^ mice.

### Ambulatory activity and metabolism

Mice were monitored in metabolic cages for a period of four days, throughout the day and the night. Activity over the last two days of the CLAMS test is represented here. *Gabrb3*^*ECKO*^ mice showed a reduced ambulatory activity versus controls specifically in the dark phase, when activity is normally increased (Fig. 3f, g). Metabolic parameters such as oxygen consumption, carbon dioxide production and respiratory exchange ratio, locomotor activity, as well as hourly food consumed and total food consumed, all were normal in the CLAMS test (Supplementary Figs. 4–6).

### Constitutive versus cell type specific contributions of *Gabrb3* determine behavioral outcome

Table 1 summarizes the differences in behaviors observed in a *Gabrb3* constitutive knockout model versus *Gabrb3* conditional knockout models in neurons or endothelial cells. Mice with a conventional knockout of *Gabrb3* in all cell types, exhibit a variety of behavioral abnormalities, such as impaired sociability, epilepsy, stereotypical behavior, poor motor skills on a repetitive task, hyperactivity, altered gamma network oscillations, learning and memory deficits, and disturbed rest-activity cycle (Table 1); phenotypic traits that were linked to autism spectrum disorder and Angelman syndrome^49,50^. Mice with a conditional (synapsin promoter-driven) knockout of *Gabrb3* in neurons^51^ in contrast, showed no seizures and normal sociability (Table 1). This knockout model was limited to pyramidal neurons of the hippocampal CA1, amygdala and cortex; however, a second neuronal-specific conditional knockout employing a CamKII promoter, showed seizure-like behavior (freezing in home cage), and impaired sociability. Learning and memory were not affected in these mice^51^.

**Table 1:** This table illustrates the similarities and differences in behavior observed in constitutive versus conditional knockout models of *Gabrb3* illustrating cell-type specific roles in behavioral diversity.

Animal models	Constitutive *Gabrb3* Knockout	Conditional *Gabrb3* Knockout; Neuron-specific	Conditional *Gabrb3* Knockout; Forebrain-specific	Conditional *Gabrb3* Knockout; endothelial cell-specific	Constitutive *Gabrb2* Knockout
Gene targeting	Targeted disruption of the β3 locus; Exons 1–3	Use of Synapsin 1-cre Target: Exon 3	Use of CamKII-cre Target: Exon 3	Use of Tie2-cre Target: Exon 3	Targeted disruption of the β2 locus; Exons 6–7
References	Homanics et al., 1997; De Lorey et al.; 1998	Ferguson et al., 2007	Ferguson et al., 2007	Li et al. 2018; Subburaju et al., 2020	Yeung et al., 2018
Time of gene inactivation	Embryonic Day 0	Embryonic Day 12.5^+^	~ 2 weeks postnatal	Embryonic Day 9^+^	Embryonic Day 0
Tissue specificity	All cells	Most neurons	Primarily forebrain neurons	Endothelial cells	All cells
Altered behaviors	Impaired cognitive functions Increased locomotor activity Hyperactive-intense circling pattern Stereotypical behavior	Cognitive functions not tested Locomotor activity not tested No hyperactivity Not reported	Normal cognitive functions Increased locomotor activity Hyper-responsive to human contact Not reported	Impaired cognitive functions-mild Increased locomotor activity Hyperactive-continuous running Stereotypical Behavior	Impaired cognitive functions Increased locomotor activity Hyperactive movement Stereotypical Behavior
Other behaviors reported	Reduced Etomidate LORR Impaired maternal behavior; foot clasping behavior EEG abnormalities, disturbed rest-activity cycle	Reduced Etomidate LORR Normal maternal behavior; no foot clasping behavior No seizure like activity observed	Reduced Etomidate LORR Impaired maternal behavior; no foot clasping behavior EEG: not tested	Increased anxiety and depression Impaired social communication and social novelty Impaired prepulse inhibition of startle	Reduced affective symptoms Impaired social affiliation and social novelty Impaired prepulse inhibition of startle
Body size	Runted until weaning	Normal	Some become obese	Runted until weaning	Normal
Neonatal lethality	90% die as neonates	61% die as neonates	30% die at P15-P25	No pup mortality	No pup mortality
Seizure-like activity	Seizures (epilepsy) Multiple seizure types	No tremors or seizures	Occasional absence-like and convulsive seizures	Seizure-like symptoms in 15% mice	Accelerated PTZ-induced seizure; susceptible to audiogenic epilepsy
Disease similarity	Angelman syndrome or autism spectrum disorder (ASD)	Unclear	ASD	Anxiety, Depression, Schizophrenia,	Schizophrenia

Interestingly, the behaviors observed in our *Gabrb3*^*ECKO*^ mice were most similar to the constitutive *Gabrb2* knockout mice, a model that has been linked to schizophrenia (Table 1). Mice with a deletion of the GABA_A_ receptor β2 subunit gene, *Gabrb2* showed behavioral dysfunction^41^ similar to schizophrenia namely a deficit in pre-pulse inhibition, locomotor hyperactivity, stereotypy, deficits in social functions, and impairment of cognitive functions (Table 1). Additionally, these knockout mice exhibit a susceptibility to audiogenic epilepsy^41^. These collective observations draw new significance as to the constitutive versus cell type specific contributions of *Gabrb3* to abnormal behaviors.

## Discussion

Anxiety, depression and schizophrenia (SZ) are multifactorial diseases characterized by complex interactions between genetic predisposition and environmental influences, and their precise cause and pathology has eluded us for more than a century. GABAergic dysfunction during the developmental period stands out as a prominent and consistently reported feature in these diseases^1,2,8–11^. Our recent studies that uncovered a novel GABA signaling pathway in CNS endothelial cells provided direct evidence for a vascular origin for psychiatric diseases^25,26^. Here, we highlight the blood flow and behavioral changes that are rooted in a dysfunction of the endothelial GABA system and find a strong connection between abnormal neocortical blood flow and behavioral dysfunction, as well as concurrent development of heart disease.

Schizophrenia is characterized by positive symptoms such as delusions, hallucinations, and disorganized thinking. Negative symptomatology can be summarized under five key areas: blunted affect, alogia, avolition, asociality, and anhedonia^52^. One of the key behavioral features of schizophrenia is a prepulse inhibition (PPI) to startle deficit, which is featured strongly in the *Gabrb2* knockout mice^41^, and our *Gabrb3*^*ECKO*^ mouse. Physiologically, the startle response consists of reflexes which are supposed to enable an individual to deal with a potentially relevant stimulus. The presentation of a weak stimulus before a strong stimulus can modulate the response, a so-called prepulse inhibition^53,54^. Our *Gabrb3*^*ECKO*^ mouse model highlights that deletion of *Gabrb3* from blood vessels is sufficient to mimic a range of symptoms, such as hyperactivity and sensorimotor gating deficits; negative symptoms such as abnormal home cage behavior, impaired sociability, as well as cognitive impairments. The *Gabrb3*^*ECKO*^ mice also show increased anxiety and depression. Taken together with the behavioral data from our earlier studies^25^, our findings suggest that the *Gabrb3*^*ECKO*^ model may serve as a useful tool for further research on anxiety, depression, and schizophrenia.

In some cases of schizophrenia psychosis, damage to the brain microvascular system has been reported, and a chronic, smoldering inflammation of the vessels has been proposed as a major contributor of the disease^31^. Fragile blood vessel pathology has been reported in people with schizophrenia, both acute and chronic. Similarly, alterations in blood flow have been reported in people with schizophrenia, but are usually interpreted as a consequence of abnormal neuronal metabolism^31^. Our *Gabrb3*^*ECKO*^ model provides the first direct evidence for a dysfunctional vascular system from early embryonic stages that persists in the adult, with impairment in several behavioral domains. Loss of endothelial *Gabrb3* caused a reduction in blood vessel densities during brain development, a time frame of active angiogenesis^25^. But loss of endothelial *Gabrb3* increased blood vessel diameters, indicating a direct effect on vessel morphology^25^. Therefore, an increase in brain blood flow was predicted in this model. The significant increase in RBC velocity and blood flow in the *Gabrb3*^*ECKO*^ neocortex that was observed in this study validated an important role for endothelial *Gabrb3* in regulating blood vessel function. The *Gabrb3*^*ECKO*^ model also helps us to better understand the cell-type specific contributions of *Gabrb3* to behavioral dysfunction. The neuronal-specific knockout of *Gabrb3* was distinct from the endothelial cell-specific knockout of *Gabrb3* in terms of altered behaviors. These endothelial versus neuronal conditional knockout models of *Gabrb3* emphasize the importance of cell-type specific GABA signaling during development as a leading contributor of the diversity seen in psychiatric symptoms.

There are deep connections between psychiatric diseases and coronary heart diseases (CHD), and there are suggestions that one may lead up to the other^55,56^. A higher prevalence of psychiatric diseases in CHD patients has been demonstrated. Conversely, people suffering from a psychiatric disease seem to have an increased risk of CHD. The precise nature of these links is not established. We observed a reduction in vascular densities in both the brain and the heart in the *Gabrb3*^*ECKO*^ model. Our study suggests that vascular *Gabrb3* has important roles in both the brain and the heart and may contribute to concurrent origin of behavioral and cardiac dysfunction. While we have characterized some of the mechanisms of this vascular GABA-GABA_A_ receptor signaling during brain development, and its direct contributions to postnatal behavior, the mechanisms of GABA-GABA_A_ receptor signaling in the heart remains unknown. It will be interesting to explore the autocrine-paracrine mechanisms of GABA_A_ receptor-GABA signaling and crosstalk between cardiac endothelial cells and cardiomyocytes in future studies. It will also be interesting to examine the long-term effects of loss of vascular *Gabrb3*-GABA signaling in cardiac tissue, and to evaluate whether this can lead up to heart failure with aging. That elimination of vascular *Gabrb3* can contribute to abnormal blood flow in the brain with downstream impairment in several behavioral domains, as well as hypertension emphasizes a novel concept that bridges a major gap between vascular biology and psychiatry.

## Methods

### Animals

Mice were housed in our institutional animal facility with a 12-h light cycle with ad libitum access to food and water. *Tie2-cre* mice and *Gabrb3 floxed* (*Gabrb3*^*fl/fl*^) mice were obtained from Jackson Labs. The *Tie2-cre* transgene is known for uniform expression of cre-recombinase in endothelial cells during embryogenesis and adulthood^57–59^. To selectively delete *Gabrb3* in endothelial cells, *Tie2-cre* transgenic mice (males) were crossed with *Gabrb3*^*fl/fl*^ mice (females) to generate *Tie2-cre; Gabrb3*^*fl*/+^ mice (males). These were further crossed with *Gabrb3*^*fl/fl*^ mice (females) to obtain the *Gabrb3* conditional knockouts (*Tie2-cre;Gabrb3*^*fl/fl*^ or *Gabrb3*^*ECKO*^ mice). Offspring stayed with their mothers until weaning. Before all behavioral testing, mice were acclimated to the testing room for 1 h. Behavioral assays were performed according to established protocols. Males were used for all behavioral assays. Experimenters scoring behaviors were blinded to the genotypes and treatment. Sample sizes for each assay are noted in figure legends. Animal experiments were in full compliance with the NIH Guide for Care and Use of Laboratory Animals and were approved by the HMRI, McLean Hospital, Massachusetts General Hospital (MGH), and Harvard Medical Area (HMA) Institutional Animal Care Committees. This study is reported in accordance with ARRIVE guidelines.

### In vivo imaging of brain microvasculature by multiphoton laser-scanning microscopy

In vivo imaging of the brain vasculature in cranial window bearing mice was performed as described previously^32,33^. Briefly, a cranial window was implanted by removing a circular area of skull and dura. Then, the window was sealed with a 7 mm cover glass glued to the bone. For the measurement of RBC velocity and blood vessel diameter, we used MPLSM^34^. To avoid potential tissue/vessel alteration caused by the window implantation procedure, we performed imaging at least 10 days after cranial window implantation. For imaging, mice were anesthetized with ketamine/xylazine, then tetramethylrhodamine (TAMRA)–dextran (MW 500,000) was systemically administrated through retro-orbital injection. Since the intravenously injected dye enhances fluorescence from the blood plasma, but not blood cells in MPLSM, RBCs appear as dark patches moving within the vessel lumen. Centerline RBC velocity was measured by repetitively scanning a line along the central axis of a single blood vessel and enabling the tracking of the motion of these dark patches. The space–time image produced by the line-scan contained diagonal dark streaks formed by moving RBCs, with a slope that was inversely proportional to the centerline RBC velocity. This space–time image was then computationally processed using MATLAB and Python to extract the gradient of each streak, corresponding to RBC velocity. This was conducted for each streak in the space–time image, and the mean gradient was taken. For the vessel diameter, an edge filter was applied to determine the blood vessel boundaries, and the blood vessel diameter extracted at the indicated region of interest. The blood flow rate was determined through the following formula:

$$\dot{{Q}} =\pi {{r}}^{2} \dot{{v}}$$

where $\dot{Q}$ is the blood flow rate, $r$ is the vessel radius, and $\dot{v}$ is the blood velocity.

### Open field locomotor activity

Mice were evaluated in locomotor activity chambers to assess spontaneous exploratory activity. The apparatus consisted of 27.3 cm × 27.3 cm × 20.3 cm, clear Plexiglas arenas equipped with infrared arrays (Med Associates; St Albans, VT, USA). Mice were placed into the center of the arena and allowed to explore freely for a total of 60 min. The mouse’s movements were tracked and recorded automatically via Med Associates software (Activity Monitor, Version 5.9) to provide measurements of total distance traveled, as well as vertical beam breaks that assess rearing behavior.

### Spatial novelty Y maze

Mice were tested in a Y maze to assess short term memory for a familiar and novel place. This test is based on the observation that mice have a preference for novelty and will prefer to explore a novel place. Testing was conducted in a clear acrylic Y maze with 3 arms (one start arm and 2 test arms, all 31 cm in length, 11 cm wide, 20 cm height), equipped with a removable partition to block the appropriate arm. The test consisted of a forced choice trial followed by a free-choice trial. For the forced choice trial, the start arm and one test arm were opened and access to the second test arm was blocked by the partition. Individual subjects were placed in the start arm and allowed to explore the open arm and start arm for 3 min, after which they were removed from the maze, and placed in a holding cage as the maze was cleaned. After 2 min, the partition was removed and the mice were placed back into the Y maze for the free choice trial and allowed to explore the start arm, familiar arm, and open novel arm for 3 min. The mouse was video recorded during both trials and the time spent in the previously accessible arm (i.e. familiar arm) and the previously blocked arm (i.e. novel arm) was measured during the free choice trial using Topscan (Version 1.0, CleverSys, Reston, VA) software. For each subject, the % distance exploring the novel arm during the free choice trial was calculated using the formula: % novel = novel/(novel + familiar time) × 100.

### Acoustic startle

The acoustic startle response is elicited by a sudden and intense acoustic stimulus that leads to a whole-body flinch. Acoustic Startle was measured using a Med Associates (St. Albans, VT) apparatus with eight chambers. Mice were placed into Plexiglas holders (4.375″ L × 1.75″ W × 1.75″ H) inside the chambers over motion sensitive transducers for detecting the startle response. After an acclimation period of 5 min, mice were given 110 trials of white noise startle pulses (40 ms duration) of various intensities (20, 30, 40, 50, 60, 70, 80, 90, 100, 110, and 120 dB) in a pseudorandom order. A variable inter-trial interval (10-20 s) was used. Startle responses for each stimulus intensity were averaged.

### Prepulse inhibition (PPI)

Prepulse inhibition (PPI) of the startle response is a phenomenon in which a short pulse of sound, presented just prior to a startle stimulus results in the dampening of the subsequent startle response. PPI was measured using the same apparatus as the acoustic startle test (Med Associates, St. Albans, VT). Mice were placed in the PPI chambers for a 5 min session of white noise (~ 65 dB) habituation followed by a habituation block of 6 presentations of the startle stimulus alone (120 dB). Following acclimation, 60 mixed trials were presented in a pseudorandom order with a variable inter-trial interval (range from 10 to 20 s): null (no stimuli), startle (120 dB), startle plus prepulse (65, 75, or 85 dB) or prepulse (85 dB) alone. The startle stimulus was a white noise startle pulse (40 ms duration) and the prepulse was a pure tone (5000 Hz, 20 ms). PPI for each prepulse intensity was calculated for each individual animal according to the following formula: PPI = (mean startle magnitude without prepulse – mean startle magnitude with prepulse)/(mean startle magnitude without prepulse/100).

### Monitoring of locomotor activity and metabolism

Columbus Instruments Comprehensive Lab Animal Monitoring System (CLAMS) was used for locomotor activity and metabolic measurements. Mice were individually housed in metabolic cages for 4 consecutive days. Mice had free access to powdered food and water ad lib and lights were on from 7 a.m.-7 p.m. Respiratory exchange ratios (RER), volumetric rate of oxygen consumption (VO_2_) and carbon dioxide production (VCO_2_) were measured by indirect calorimetry using CLAMS. Ambulatory activity (Xamb) and rearing (Z counts) were collected by infrared beam breaks. The CalR software^60^ was used to analyze the CLAMS data. Data are presented for the last 48 h (7 a.m.-7 a.m.) of the 4-day period.

### Cardiac characterization

Blood pressure measurements were conducted with the well-established tail cuff method^39^. For PSR and H&E staining, animals were anesthetized with xylazine and ketamine i.p. injection. An abdominal incision was made to expose the aorta and inferior vena cava. A catheter was placed in the abdominal aorta positioned towards the heart for perfusion fixation and a nick was made in the inferior vena cava to drain the blood. Phosphate buffer solution was injected for 3 min at a pressure equal to the mean blood pressure (122 cm H2O, 90 mm Hg) to remove blood; thereafter KCl was injected to arrest the heart in a diastolic phase followed by 15 min perfusion with Zinc fixative (BD Biosciences). The fixed heart was excised and immersed in Zinc fixative for further fixation, processing and histology.

### Statistical analysis

For each experiment, we used either 1 or 2 mice from a given litter. For blood flow experiments and cardiac characterization, 5–7 litters of mice were used. For behavioral experiments, 10-14 litters of mice were used. Statistical significance of differences between groups was analyzed by either two-tailed Student's *t* test or ANOVA (Prism; GraphPad software) and has been noted in individual figure legends. The measured variables followed the normal distribution in the Shapiro–Wilk test. Significance was reported at *P* < 0.05.

## Supplementary Information

Supplementary Information.
