# Supplementary material for: Gabrb3 endothelial cell-specific knockout mice display abnormal blood flow, hypertension, and behavioral dysfunction
Source: Sci Rep. 2022 Mar 22;12:4922. doi: 10.1038/s41598-022-08806-9 (PMC8941104; doi:10.1038/s41598-022-08806-9)
Supplement: Supplementary file 1 — Supplementary Information. [file 41598_2022_8806_MOESM1_ESM.pdf]

Supplementary Figure 1

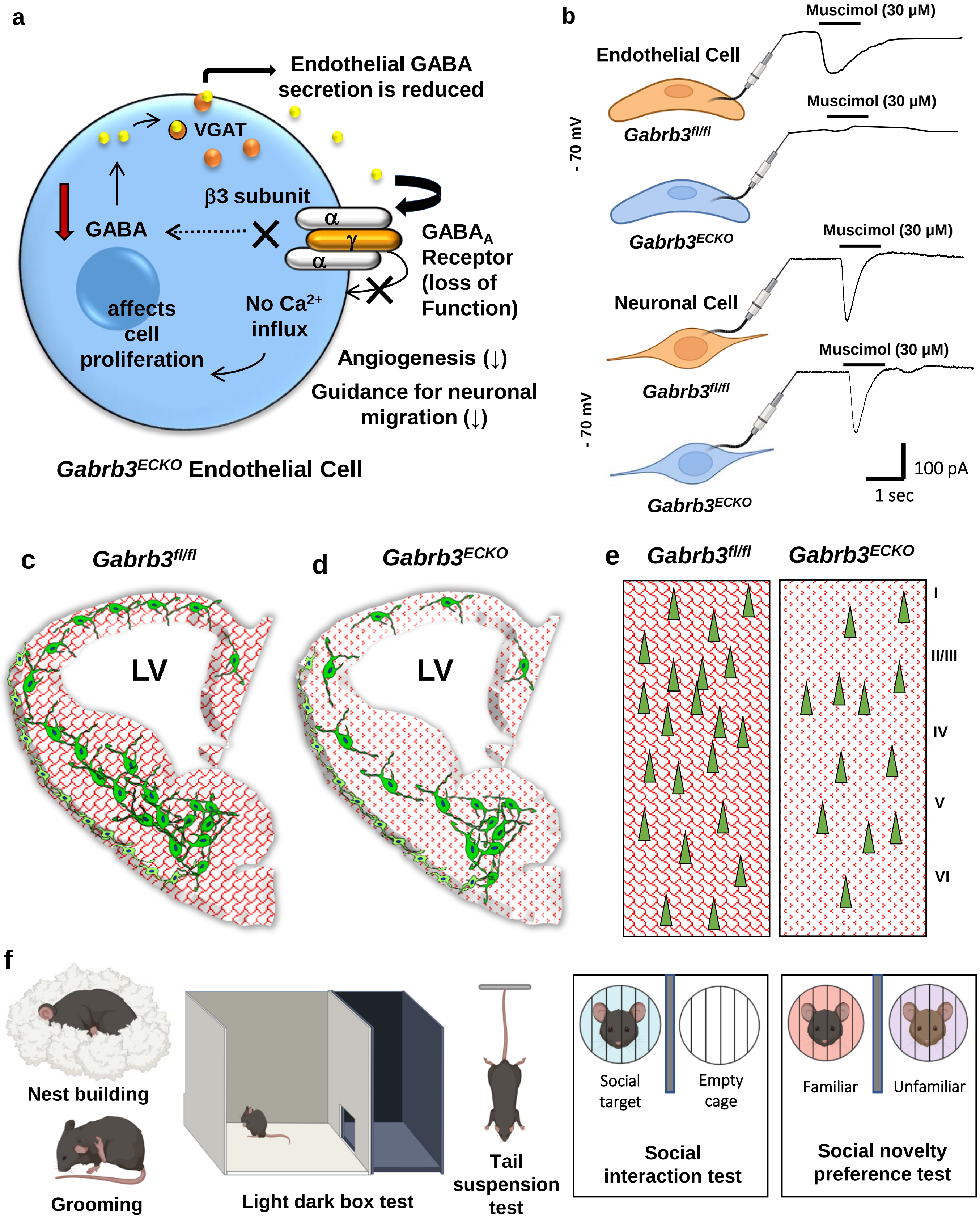

Behavioral assays that showed abnormalities in *Gabrb3^{ECKO}* mice

Supplementary Figure 1: The significance of endothelial *Gabrb3* for brain development and behavior.

(a) Schema depicting defects in the positive feedback GABA signaling pathway in *Gabrb3*^{ECKO} endothelial cells, in which due to loss of the β3 subunit, $GABA_A$ receptors become dysfunctional. As a result, endothelial cell-derived GABA is unable to activate $GABA_A$ receptors and cannot trigger Ca^{2+} influx, and endothelial cell proliferation. GABA expression is also downregulated in *Gabrb3*^{ECKO} endothelial cells. This affects GABA secretion from *Gabrb3*^{ECKO} endothelial cells and disturbs paracrine GABA signaling for neuronal migration, and autocrine GABA signaling for angiogenesis. (b) Control endothelial cells have functional $GABA_A$ receptors that account for the GABA responses, while there was no current response in *Gabrb3*^{ECKO} endothelial cells, indicative of loss of function. There was no change in the $GABA_A$ receptor response in cortical neuronal cells from *Gabrb3*^{ECKO} telencephalon that emphasized the specific loss of $GABA_A$ receptor function in endothelial cells. (c) *Gabrb3*$^{fl/fl}$ telencephalon with normal vascular network (red lattice pattern) promotes tangential GABAergic neuronal migration (green) from the ventral telencephalon. (d) In *Gabrb3*^{ECKO} telencephalon, due to dysfunctional endothelial $GABA_A$ receptors and partial loss of endothelial GABA secretion, both angiogenesis (intricate red pattern) and GABAergic neuronal migration are reduced. (e) A reduction in blood vessel densities and GABAergic interneurons were observed in cingulate, motor, somatosensory, and piriform cortex of *Gabrb3*^{ECKO} mice at P90, when compared to *Gabrb3*$^{fl/fl}$ mice. (f) Several behavioral deficits were observed in the *Gabrb3*^{ECKO} mice in assays such as nest building, grooming, light-dark box test, tail suspension test, social interaction test, and social novelty preference test.

Supplementary Figure 2

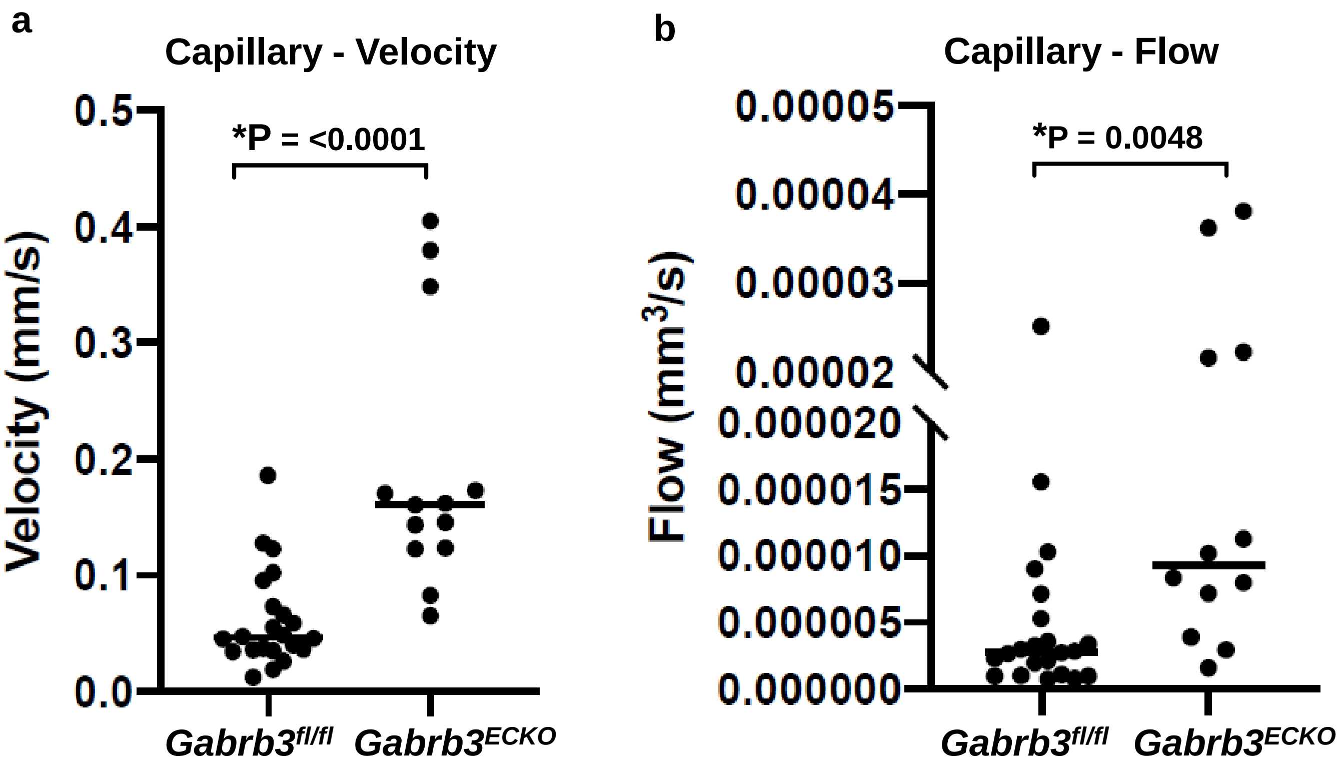

Supplementary Figure 2: Blood flow changes in *Gabrb3*^{ECKO} mice.

(a) Dot plot of the violin plot in Figure 1b with its individual data points, that shows the distribution of RBC velocities in capillaries (n = 22, n = 13 vessels, respectively).

(b) Dot plot of the violin plot in Figure 1c with its individual data points, that shows the distribution of blood flow in capillaries of *Gabrb3*$^{fl/fl}$ and *Gabrb3*^{ECKO} mice (n = 22, n = 12 vessels, respectively).

Supplementary Figure 3

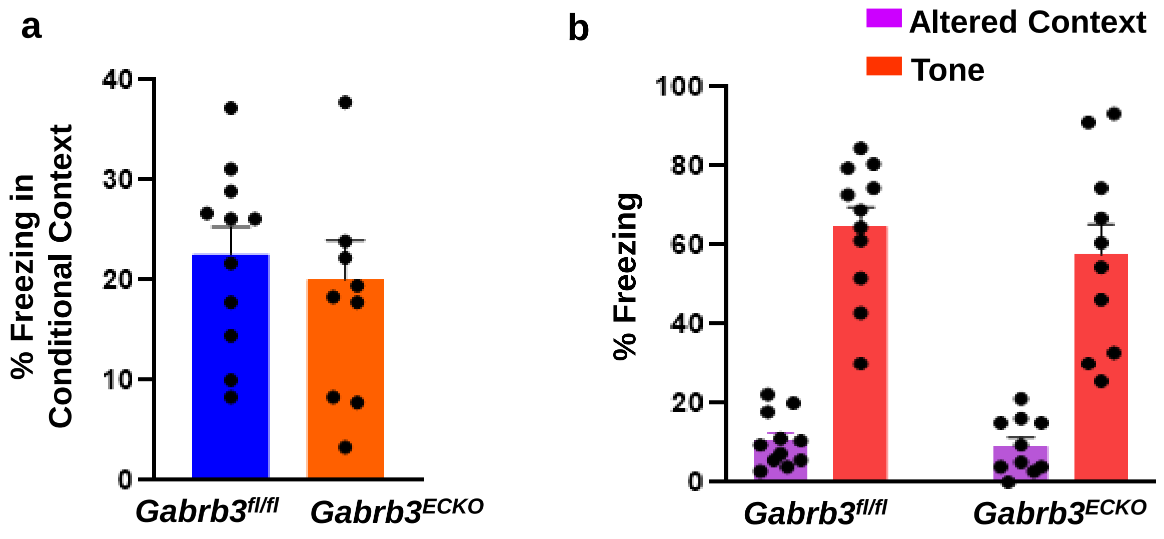

Supplementary Figure 3: Contextual and Cued Fear Conditioning.

This paradigm is widely used to assess hippocampal and amygdala-dependent learning and memory performance in rodents**.** The test is based on the tendency of mice to show a fear response (freezing) when re-exposed to the context and discrete tone paired with an aversive stimulus (foot shock). Mice were exposed to a conditioning chamber (31 cm-L x 25 cm-W x 25 cm-H, Med Associates) with Plexiglass sidewalls, stainless steel end-walls, and a floor consisting of steel bars. On the first day (training), mice were allowed to explore the chamber for 2 min and then were given a 30 sec tone that terminated in a 2 sec foot shock (0.5mA). After 2 min, mice were given a second 30 sec tone terminating in a 2 sec foot shock. Mice were removed from the chamber 30 sec after the last foot shock. Approximately 24h after training, mice were exposed to the conditioning chamber for 5 min (no electric shock was delivered during this session), freezing was recorded and the first 3 min of the test session was scored. The freezing response is used as a surrogate marker of memory performance as mice remembering receiving the shock during the training session are expected to spend a significant amount of time freezing during the retention session. Following the contextual conditioning test, the context was altered by covering the shock grid, and walls and cleaning the chamber with a different odor. Mice were tested for freezing behavior in this altered context for 3 min. Mice should show less freezing in the altered context if they can discriminate the contextual from the altered context. The tone paired with the shock was then played continuously for 3 min and freezing was scored during this time. Mice should show increased levels of freezing during the tone if they associate the pairing of the tone with foot shock. No differences were observed in contextual and cued fear conditioning in the *Gabrb3^{ECKO}* mice versus the *Gabrb3$^{fl/fl}$* mice (a, b).

Supplementary Figure 4

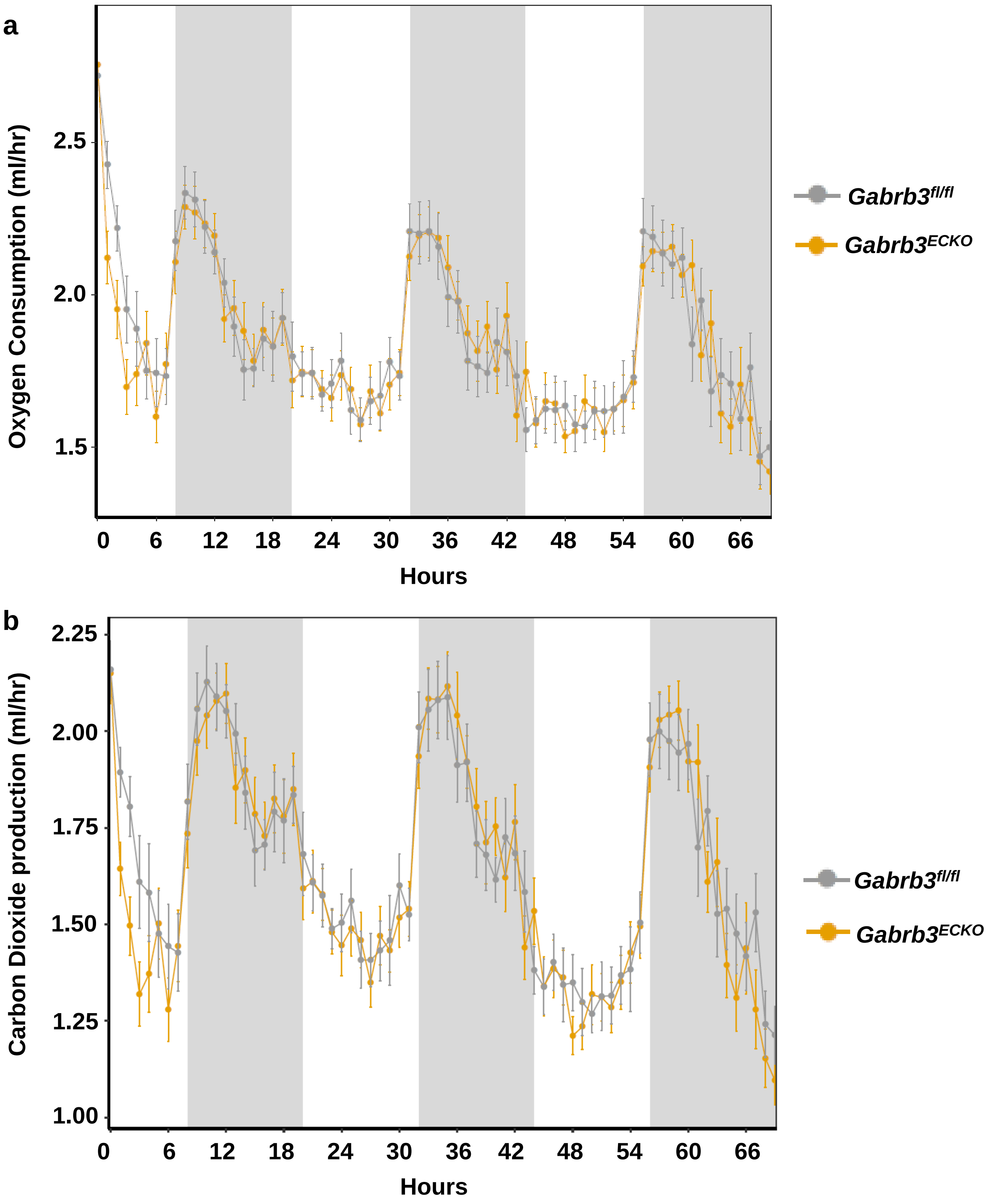

Supplementary Figure 4: Metabolism.
No changes were observed in oxygen consumption (a) and carbon dioxide production (b) between *Gabrb3*$^{fl/fl}$ and *Gabrb3*^{ECKO} mice in the CLAMS test.

Supplementary Figure 5

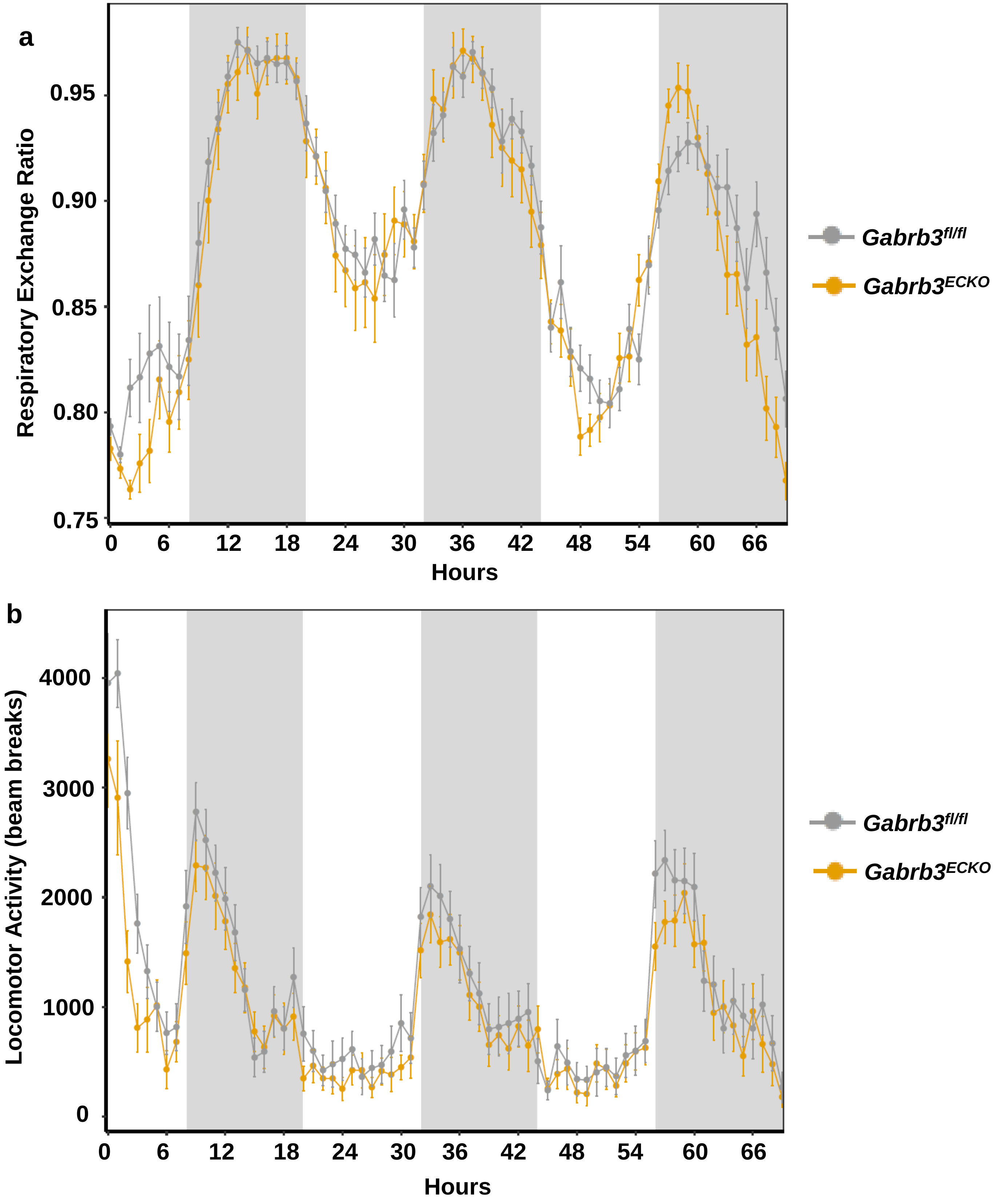

Supplementary Figure 5: Metabolism and Locomotor Activity.
No changes were observed in the respiratory exchange ratio (a) and in the locomotor activity (b) between *Gabrb3*$^{fl/fl}$ and *Gabrb3*^{ECKO} mice in the CLAMS test.

Supplementary Figure 6

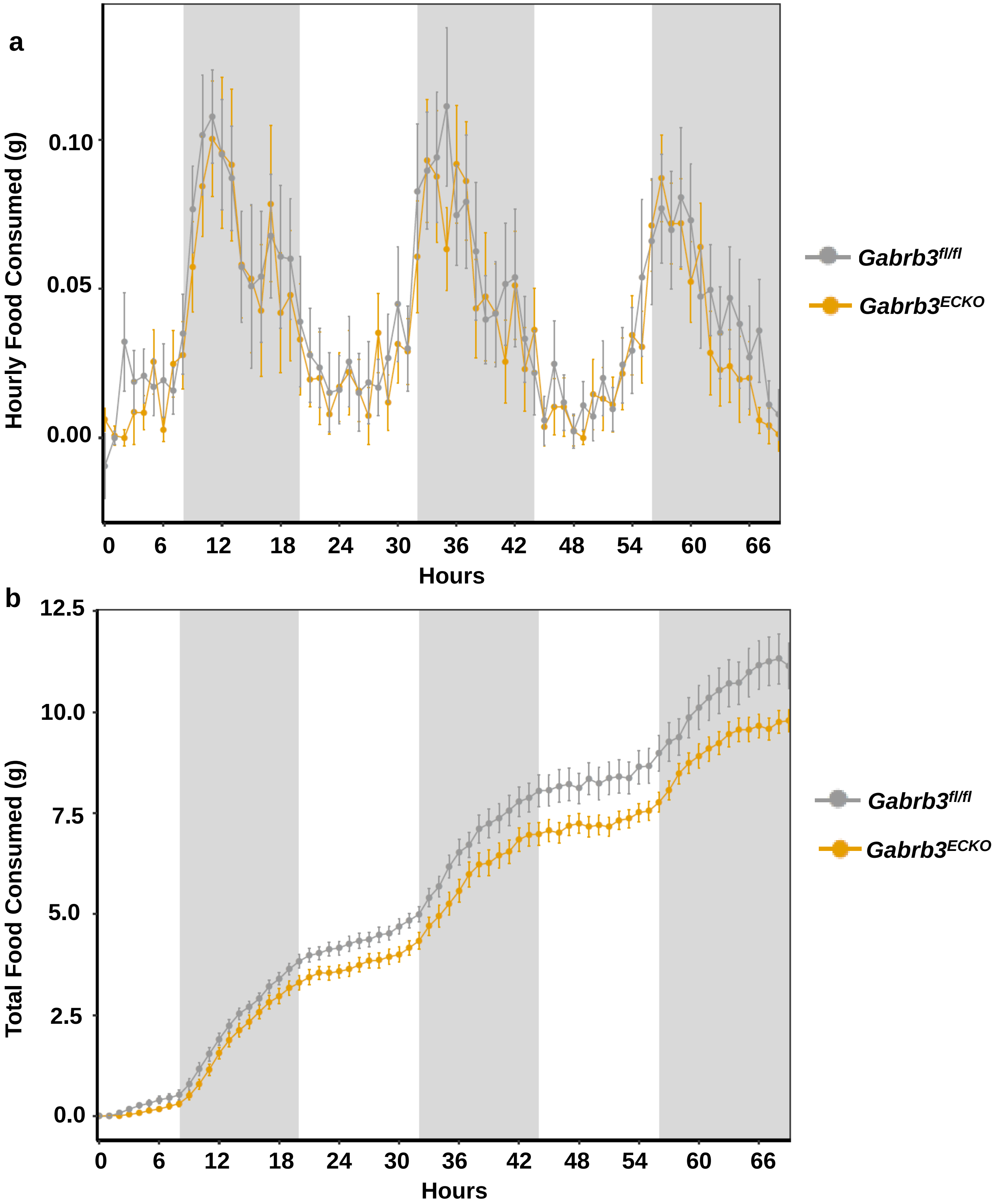

Supplementary Figure 6: Food Consumed.

No changes were observed in the hourly food consumed (a) and the total food consumed (b) between *Gabrb3*$^{fl/fl}$ and *Gabrb3*^{ECKO} mice in the CLAMS test.
